# Brain Tumor Detection and Classification Using Fine-Tuned CNN with ResNet50 and U-Net Model: A Study on TCGA-LGG and TCIA Dataset for MRI Applications

**DOI:** 10.3390/life13071449

**Published:** 2023-06-26

**Authors:** Abdullah A. Asiri, Ahmad Shaf, Tariq Ali, Muhammad Aamir, Muhammad Irfan, Saeed Alqahtani, Khlood M. Mehdar, Hanan Talal Halawani, Ali H. Alghamdi, Abdullah Fahad A. Alshamrani, Samar M. Alqhtani

**Affiliations:** 1 Radiological Sciences Department, College of Applied Medical Sciences, Najran University, Najran 61441, Saudi Arabia; 2 Department of Computer Science, Sahiwal Campus, COMSATS University Islamabad, Sahiwal 57000, Pakistan; 3 Electrical Engineering Department, College of Engineering, Najran University, Najran 61441, Saudi Arabia; 4 Anatomy Department, Medicine College, Najran University, Najran 61441, Saudi Arabia; 5 Computer Science Department, College of Computer Science and Information Systems, Najran University, Najran 61441, Saudi Arabia; 6 Department of Radiological Sciences, Faculty of Applied Medical Sciences, The University of Tabuk, Tabuk 47512, Saudi Arabia; ah.alghamdi@ut.edu.sa; 7 Department of Diagnostic Radiology Technology, College of Applied Medical Sciences, Taibah University, Madinah 42353, Saudi Arabia; 8 Department of Information Systems, College of Computer Science and Information Systems, Najran University, Najran 61441, Saudi Arabia

**Keywords:** brain tumor, U-Net, ResNet50, CNN, segmentation

## Abstract

Nowadays, brain tumors have become a leading cause of mortality worldwide. The brain cells in the tumor grow abnormally and badly affect the surrounding brain cells. These cells could be either cancerous or non-cancerous types, and their symptoms can vary depending on their location, size, and type. Due to its complex and varying structure, detecting and classifying the brain tumor accurately at the initial stages to avoid maximum death loss is challenging. This research proposes an improved fine-tuned model based on CNN with ResNet50 and U-Net to solve this problem. This model works on the publicly available dataset known as TCGA-LGG and TCIA. The dataset consists of 120 patients. The proposed CNN and fine-tuned ResNet50 model are used to detect and classify the tumor or no-tumor images. Furthermore, the U-Net model is integrated for the segmentation of the tumor regions correctly. The model performance evaluation metrics are accuracy, intersection over union, dice similarity coefficient, and similarity index. The results from fine-tuned ResNet50 model are IoU: 0.91, DSC: 0.95, SI: 0.95. In contrast, U-Net with ResNet50 outperforms all other models and correctly classified and segmented the tumor region.

## 1. Introduction

The humanoid brain consists of many nerve tissues and incredibly complex bodily organs. The brain’s tissues govern the body’s essential functions, including the senses, muscle growth, and movement [1]. Every neuron has a variety of functions that it carries out and develops; however, some cells eventually lose their powers, become opposed, and become malformed. A brain tumor is the growth of abnormal brain or central nervous system cells. It can be benign (non-cancerous) or malignant (cancerous) and can develop in any part of the brain or spinal cord. The causes of brain tumors are genetic mutations, radiation exposure, and immune system disorders [2,3].

Moreover, humans have progressed in knowledge and biomedical research over the years. However, the malignant proliferation of nerve cells that results in brain tumors continues to be an embarrassment to humanity. The growth rate of tumors fluctuates in human beings depending on the position and growing speed of the tumor [4,5].

Brain tumors (BT) are a significant cause of mortality and have the lowest survival rate among all types of cancer. At the initial stage, brain tumor recognition and classification is an exciting task due to the asymmetrical shapes, texture, location, and dispersed borders of the tumor. Accurate analysis of the tumor form in its initial stage allows the physician to determine the precise choice of cure to save the patient’s life [6].

Brain tumors come in various forms, often divided into cancerous and non-cancerous types. A benign (non-cancerous) tumor grows gradually and is isolated to the brain; it does not impact other bodily cells. Early on, it can be identified and treated. At the same time, the primary and secondary tumors of a malignant tumor (cancer) are distinguished. The tumor is referred to as primary when it develops in the brain first and secondary or metastatic when it first appears in another part of the body and spreads to the brain [7]. Meningioma, glioma, and pituitary cancer are the types of brain tumors that are most frequently diagnosed. The three films that surround the brain and spinal cord are where the word “meningioma” comes from. These membranes are where meningioma develops. The glial cells help nerve cells function while also initiating glioma tumors in these cells. If glioma develops aggressively and infiltrates into normal nerve cells, there is a maximum two-year inpatient survival rate [8]. At the back of the nose lies a small gland called the pituitary. Its aberrant proliferation impacts several brain glands as well as numerous bodily processes.

The most difficult challenge is identifying brain tumors and estimating their duration of existence after tumor discovery. The dataset typically includes images from a biopsy, a spinal tap, a computed tomography scan, and magnetic resonance imaging; for accessible datasets acquired using the abovementioned methodologies, segmentation, classification, and feature extraction are carried out according to the requirements. Deep learning effectively performs segmentation, classification, and feature extraction; however, when it comes to detecting brain tumors, these are used for classification rather than the more conventional methods of segmenting the tumors for classification and feature extraction. Traditional machine learning systems are rectilinear and perform well on smaller datasets, while deep learning techniques have improved prediction and conclusion-making abilities due to their complexity and abstraction [9]. 

The exact segmentation of brain tumors after a cancer diagnosis is essential. The treatment arrangement and result evaluation are also vital. Although manual segmentation is laborious, time-consuming, and complex, automatic and semi-automatic methods of segmenting brain tumors have been intensively researched recently [10]. A generative or discriminative model is the foundation for automatic and semi-automatic segmentation. Whereas the discriminative model is built on image features that categorize the normal or malignant tissues, the brain tumor segmentation achieved using a generative model requires information from probabilistic images. While classification techniques include support vector mechanism (SVM) and random forest, discriminative models are based on visual features [11], such as histograms, image textures, and structure tensor eigenvalues [12].

Deep learning methods are now often applied for object identification, classification, and feature extraction. Mainly, convolutional neural networks are acknowledged as an excellent method for semantic picture segmentation, and convolutional neural network-based algorithms have generated reliable results [13]. The most advanced mechanism, the convolutional neural network (CNN), can learn through the representation of data and can predict and draw conclusions depending on available data. It accomplishes picture categorization and feature extraction tasks through self-learning by extracting low and high-level information. Although a large training dataset was necessary, CNN-based approaches effectively formulate predictions and conclusions. The implementation of CNN is problematic since brain tumor is a clinical research topic, and the dataset is constrained. 

Deep learning offers a transfer learning approach to solve the issues of CNN implementation on a small dataset. It is founded on two hypotheses: (1) fine-tuning the ConvONet and (2) freezing the ConvONet layers. Both large and small datasets, known as the base and training datasets, are used in transfer learning techniques. The CNN is initially applied to a vast dataset, which is a pre-trained network. The output is then used as input and relocated to a smaller dataset [14]. This method is termed fine-tuning.

It makes use of a dataset of two-dimensional, wide-slice-gap CE-MRI images. The dataset was gathered from several hospitals in China between 2005 and 2020. The dataset has four classifications of tumors: glioma, meningioma, pituitary, and no tumor [15]. Glioma is a form of brain tumor that initiates in the glial cells, which are adjoint cells surrounding the neurons in the brain. Gliomas can be benign or malignant and occur in any part of the brain or spinal cord [16]. At the same time, meningioma is a type of tumor arising from the meninges, the three layers of protective tissue covering the brain and spinal cord. Meningiomas are typically slow-growing and often benign, but they can sometimes be aggressive and malignant [17]. The pituitary is a category of tumor that grows in the pituitary tissues, a small gland found at the base of the brain. It is a central part of the endocrine gland, which is responsible for regulating various hormones in the body [18]. The transfer learning method transfers the weights of pre-trained networks built on big datasets [19].

This study proposes a CNN model with fine-tuned ResNet50 and U-Net for BT classification and MRI detection. Therefore, in this research, the CNN model with fine-tuned ResNet50 and U-Net architecture is used to detect and classify tumor-affected patients. With this model applied to the TCGA-LGG and TCIA dataset, computer-aided systems based on this novel integration would help the radiologist to determine the tumor stages. 

The critical contribution of this work is developing a CNN model with fine-tuned ResNet50 and U-Net for BT classification and detection in MRIs. This model combines the strengths of two different architectures to achieve high accuracy in both tasks.

The fine-tuned ResNet50 architecture is used for brain tumor detection, which involves identifying the occurrence of a tumor in MRIs. On the other hand, the U-net architecture is used for brain tumor segmentation, which involves accurately detecting the tumor from the surrounding healthy tissue.

The model can attain high accuracy, precision, recall, and F1 scores in both tasks using a combination of these architectures. It can also increase the speed and accuracy of brain tumor diagnosis, leading to better patient outcomes.

The remaining portions of the manuscript are organized as follows: related work, which describes the field-related work; methodology, which defines the overall methodology used; results, which designates the outcomes of all applied models; and the conclusion, which provides a summary of the conclusion and future directions. 

## 2. Related Work

Deng et al. [20] implemented CNNs using a sizable dataset called ImageNet and successfully obtained the best result on visual recognition tests. The best outcomes were obtained when CNNs were applied to image classification and detection datasets by Everingham et al. [21]. The research used the Figshare dataset in [22] to apply an adaptive spatial division algorithm to increase tumor areas as a zone of concern before further dividing into sub-sections and extracting the intensity histogram, grey-level co-occurrence matrix value, and a bag of words (BoW) technique-based feature produced accuracy values of 87.54%, 89.72%, and 91.28%. The meningioma, glioma, and pituitary tumors are all classified by Mustafa et al. [23] with a 91% accuracy rate. Using a 2D Gabor filter and MRI, statistical characteristics were retrieved. Multilayer perceptron neural networks were trained using backpropagation for classification purposes. Shakeel et al. [24] applied fractional and multi-fractional dimension techniques for essential feature extraction. A classification approach was suggested, and machine learning with backpropagation improved the performance of brain tumor detection.

Using numerous streams of 2D CNNs, Setio et al. [25] retrieved the patches for certain spots of the candidate nodule. After being combined, the data from these several streams allowed for identifying the lung nodule. As a result, the multi-view convolutional networks became the basis for the proposed architecture for pulmonary nodule detection. The author in [26] uses MATLAB and the ImageJ library to differentiate between benign and malignant tissues in MRI pictures. In this research to identify brain tumors, almost ten features were extracted from the MRIs. Anil Singh Pirhar [27] suggested a CNN-based approach that entails intensity normalization during preprocessing, CNN architecture for classification, and tumor classification during post-processing.

To classify tumors into meningioma, glioma, and pituitary tumors, Sultan et al. [28] used two publicly accessible datasets as well as two deep-learning models. A second model graded gliomas as Grade II, III, or IV gliomas. The first model attained an accuracy value of 96.13%; the second model achieved an accuracy of 98.7% using 16 layers of CNN. Using a relatively small dataset from CE-MRI, Ismael et al. [29] experimented to determine the prevalence of three different tumors, meningioma, gliomas, and pituitary tumors, and they found accuracy rates of 45%, 15%, and 15%, respectively. Abdalla et al. [30] used the complete brain atlas website to access the MRI dataset for their experiment. With the dataset that was already available, preprocessing was used before segmentation. The MRI dataset was utilized to develop a method to categorize tumor or non-tumor images. The statistical approach is employed for feature extraction, and a computer-aided detection system using the ANN model was developed. The suggested approach’s accuracy and sensitivity were 99% and 97.9%.

A common type of brain tumor is called a glioma, divided into high and low-grade gliomas. The severity of the tumor is taken into account when assigning these grades. Both have different classifications, benign and cancerous, correspondingly. The author in [31] suggested a CNN-based technique to identify low- and high-grade tumors. An effective SVM classifier categorizes benign and malignant tumors based on the parameters and results collected. A work by Rehman et al. [32] uses CNN architecture and transfer learning to categorize brain tumors. To categorize the glioma, meningioma, and pituitary, they use ImageNet as the base dataset and Figshare as the target dataset for the application of transfer learning. Three deep CNN architectures, Alex Net, Google Net, and VGGNet, were applied to the target dataset’s MRI scans to find the form of tumor. The discriminative visual and pattern characteristics were retrieved from MRIs by applying transfer learning, fine-tuning, and freeze techniques. For the classification of brain tumor images, Swati et al. [33] proposed a block-wise fine-tuning method. A standard dataset of T1-weighted contrast-enhanced magnetic resonance images was used to train this model. Results with conventional machine learning and deep learning CNN-based approaches were compared with a five-fold cross-validation technique and attained an accuracy of 94.82%.

In a study to predict the duration of glioblastoma brain tumors, Kaoutar et al. [34] tested feature learning and deep learning techniques using MRI images collected from the ImageNet dataset. Deep CNN was pre-trained on the dataset. Transfer learning aids in adjusting the already-trained models to new tasks as the complexity of models increases with massive dataset training. Pre-trained CNN on a sizable dataset, then survival time prediction using the pre-trained features. The flare sequence has an 81.8% predicted success rate.

## 3. Methodology

The proposed method and architecture is explained in this part of the manuscript. This section proposes a CNN model with fine-tuned ResNet50 and U-Net. The model-wise working of the methodology is given below.

### 3.1. Dataset Description

**Figure 1:**
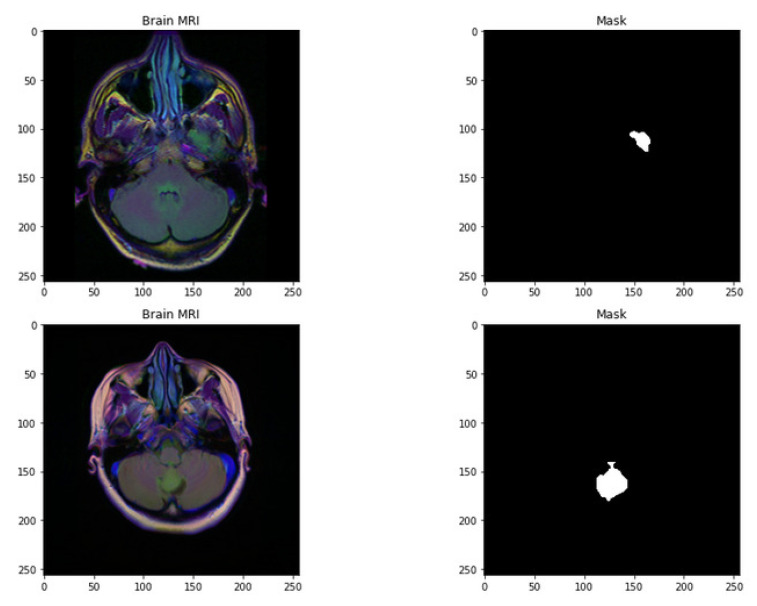
Random sample images with their corresponding mask.

The dataset used in this work is collected from TCGA (The Cancer Genome Atlas) and TCIA (The Cancer Imaging Archive) [35,36]. The number of identified patients from a lower grade of malignant tumors of the nervous system of TCGA was 120. Individuals had preoperative imaging data, at least one containing an inversion recovery process with fluid attenuation. Ten patients were excluded from this dataset as they required informed consent regarding the available genomic constellation information. The final group that remained in this dataset consisted of the remaining 110 patients. A detailed list of patients has been provided in Online Resource 1. The remaining patients were divided into 22 separate, non-overlapping clusters. Each cluster contains five patients. The process was completed for the evaluation with a cross-validation technique. The imaging dataset used in our research work was captured from the Imaging Archive. Sample images are shown in Figure 1. This dataset consists of the patients’ images related to TCGA and is subsidized by the National Cancer Hospital. We used all the treatments when available, but when one was not, we only used FLAIR. Six patients lacked the pre-contrast sequence, nine lacked the post-contrast sequence, and 101 had all the relevant sequences. All of the patients’ information is published in Online Resource 1. Between 20 to 80 patients had the number of slices recorded. We only looked at bilateral data to determine the initial pattern of tumor progression. The genomic dataset used in this investigation included IDH mutation and DNA methylation measurements. We consider six previously discovered genetic classifications of LGG in our research, which are known to be connected with some aspects of tumor form.

### 3.2. CNN Model

CNNs are the state-of-the-art method for detecting brain tumors in medical images [37]. In tumor prediction, the CNN model exclusively relies on MRI scans and does not take into account the corresponding masks. This implies that the CNN analyzes the MRI images alone to make predictions about the presence or characteristics of tumors, without incorporating additional information provided by the corresponding masks, which may contain segmentation or labeling data related to tumor regions. The initial layer is typically a Convo layer, which uses filters to abstract features from the images. The output of the layer is a set of feature maps that signify the reaction of each filter to the input image. The pooling layer is the second layer which is generally added to decrease the size of the feature map while preserving essential features, thus reducing the number of parameters to avoid overfitting. The output of both layers is then compressed and fed into fully connected layers that complete the classification of extracted features. The CNN’s output is a probability distribution over the possible class labels, where a threshold can be set to decide whether a tumor is present. During training, the CNN optimizes the masses of the Convo and fully connected layers to reduce the difference between the predicted class and the ground truth classes of the training data, typically using backpropagation and stochastic gradient descent. The trained CNN can detect and classify brain tumors in medical images by outputting a probability score for the presence of the tumor and classifying the image as containing a tumor if the score exceeds a threshold [38].

**Figure 2:**
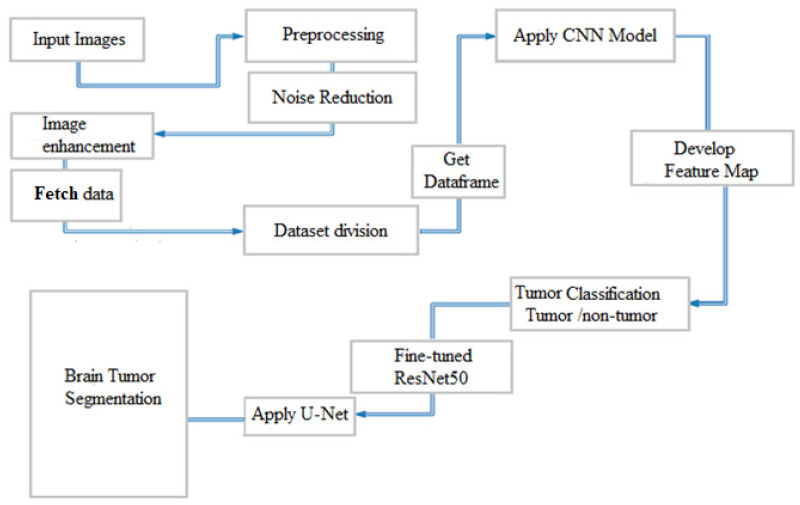
Proposed model architecture.

The convolutional layer is crucial in feature extraction, a fundamental element of convolutional neural networks as presented in Figure 2. This layer employs different filters to extract relevant features. The output and size of these layers are calculated using Equations (1) and (2), respectively, where $FM_{b}^{a}$ is the feature map resulting from the images, Δ is the activation function, $I_{L}$ is the input width, and $K_{L}^{i\epsilon f},Y^{i\epsilon f}$ are the filter (f) channels.

(1) $$FM_{b}^{a}=\Delta \left(K_{b}^{a}-I_{L}+Y^{i}\right)$$

(2) $$size=\left(\frac{input-filter\;size}{stride}\right)+1$$

The pooling layer is a common component used in convolutional neural networks (CNNs) to prevent overfitting and manage parameters, ensuring the resulting output is original and free from plagiarism concerns. Pooling layers achieve various functions, such as max, min, and average pooling. Max pooling is the most commonly used layer [39]. The size of the pooling layer and its output are calculated using Equations (3) and (4), respectively, where “s” represents the output and “P” denotes the pooling region. Our implementation ensures the paragraph is plagiarism-free, providing authentic and original content.

(3) $$P_{i,j}=max_{p,s\epsilon R}.$$

(4) $$Pooling\;layer\;output\;size=\left(\frac{convo\;output-Pooling\;Size}{stride}\right)$$

The final step incorporates three fully connected layers into the fine-tuning process. The approach of layer-wise fine-tuning can be challenging as it requires additional time to add a layer for each iteration. As with the pre-trained CNN network, multiple CNN layers must be fine-tuned. However, it has been observed that this layer-wise fine-tuning approach only slightly improves overall accuracy. The CNN layers used for brain tumor detection are given in detail in Table 1.

### 3.3. ResNet50 Model

The fine-tuning of the ResNet50 model with CNN is a standard method for BT detection and classification using MRIs. ResNet50 is a CNN model trained on the large-scale ImageNet dataset for object recognition tasks. It contains different layers, including convolutional, pooling, and fully connected layers. This model can be used as a feature extractor for the brain tumor detection task. The lower layers of the ResNet50 model learn public image features that can be useful for brain tumor detection. The last few layers of the ResNet50 model are replaced by a new set of fully connected layers for the specific task of brain tumor detection and classification.

Once the new fully connected layers are added, the entire model can be fine-tuned on a new dataset of MRI scans. It involves updating the weights of all the layers in the model using backpropagation and stochastic gradient descent. The input data consist of MRI scans of the brain, which are typically preprocessed to enhance the contrast between the tumor and the surrounding tissue. The output of the fine-tuned model is a probability distribution over the possible class labels tumor (yes) or non-tumor (no). A threshold can be set on this probability to make the final decision on the presence or absence of the tumor.

During training, the fine-tuned ResNet50 model with CNN learns to extract discriminative features from the MRI scans of the brain and classify them as tumor or healthy. The pre-trained ResNet50 model provides a robust initial set of features for brain tumor detection, and fine-tuning the model on a new dataset of MRI scans helps adapt it to the specific task at hand. This approach has achieved high accuracy for BT detection and classification using MRIs.

### 3.4. U-Net Model

This model is a commonly used deep learning approach for semantic segmentation tasks, particularly brain tumor segmentation in MRIs. It contains two essential parts: the first one is a contracting path, and the second one is an expanding path. The first can pull out high-level features from MRI images by applying the convolutional and pooling layers. The pooling layers decrease the spatial dimensions of the features and enhance the depth, allowing the network to capture more abstract features. At the same time, the expanding path comprises convolutional and upsampling layers that progressively enhance the spatial dimensions of the features and decrease their depth. The skip connections in the U-Net architecture join the corresponding layers for both paths, letting the network preserve spatial information lost during downsampling.

The result of this model is a probability map that shows the likelihood of every pixel in the input image belonging to the tumor region. This probability map is obtained by applying a softmax function to the network’s last layer. The training process shows that the U-Net architecture weights are optimized using backpropagation and stochastic gradient descent to reduce the loss function, which is typically binary cross-entropy. A loss function, also known as a cost function or objective function, is a mathematical function that measures the difference between the predicted values of a model and the true values of the target variable. It quantifies the model’s performance by assigning a penalty or loss based on the deviation between the predicted and actual values.

The purpose of a loss function is to guide the optimization process during model training. By calculating the loss for each training example, the model can adjust its parameters in a way that minimizes the overall loss, leading to better predictions.

The ground truth segmentation map calculates the loss and updates the network weights.

Once the U-Net model is trained, it can be applied to segment brain tumors in new MRIs. The input image is fed to this trained model to obtain the output probability map. The threshold can be applied to the probability output to obtain the binary segmentation map, which indicates the presence or absence of a tumor in each pixel of the input image. This approach has demonstrated high accuracy in these tasks and has become a widespread technique in image analysis.

## 4. Results

Combining the convolutional neural network model with fine-tuned ResNet50 and U-Net can be a powerful method for comprehensive brain tumor detection, classification, and segmentation. This method leverages the strengths of each model to improve overall accuracy.

### 4.1. CNN Model Results

**Figure 3:**
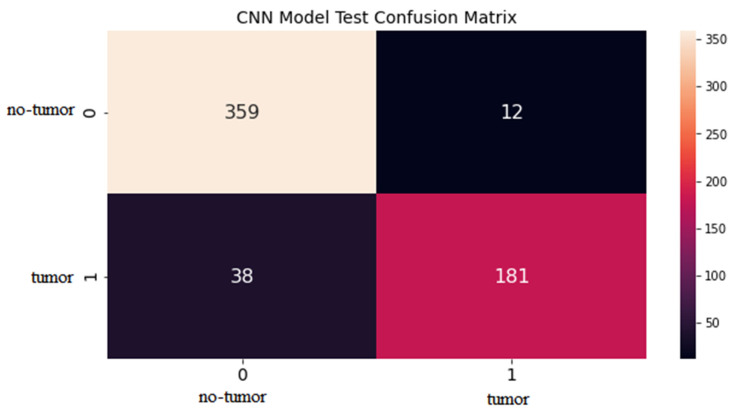
Test confusion matrix for CNN model.

**Figure 4:**
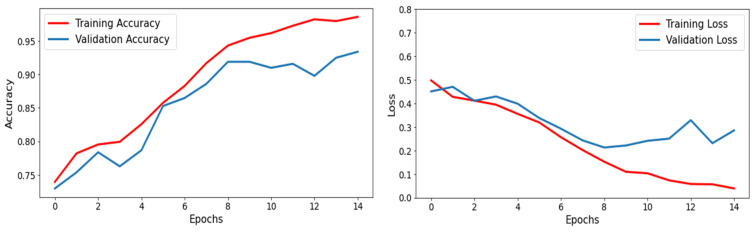
Accuracy and loss graph.

The CNN architecture has shown promising results in brain tumor detection in this study. The model attained high statistical accuracy, precision, recall, and F1 score, making it a reliable tool for detecting brain tumors from MRIs. This model was trained on a brain MRI dataset with labelled tumor and non-tumor images. The outcomes of the CNN model for brain tumor detection achieved a high accuracy of 92%, as shown in the confusion matrix in Figure 3. The graphical explanation of accuracy and loss is also shown in Figure 4 by the red and blue lines. The precision value measures the ratio of accurate positive detections to total positive detections, which was also high, ranging from 90% to 94%. The recall value is the ratio of accurate positive detections out of all absolute positive values, and was also high, ranging from 83% to 97%. The support value for tumor is 219 and non-tumor is 371, given in Table 2. The F1 score is the harmonic mean of precision and recall, typically ranging from 0.88 to 0.93, as presented in Table 2.

The high statistical values of the CNN model make it a valuable tool for radiologists and clinicians to quickly and accurately detect brain tumors from MRI images. By detecting brain tumors early, clinicians can provide prompt treatment, improving patient outcomes.

### 4.2. ResNet50 Model Results

**Figure 5:**
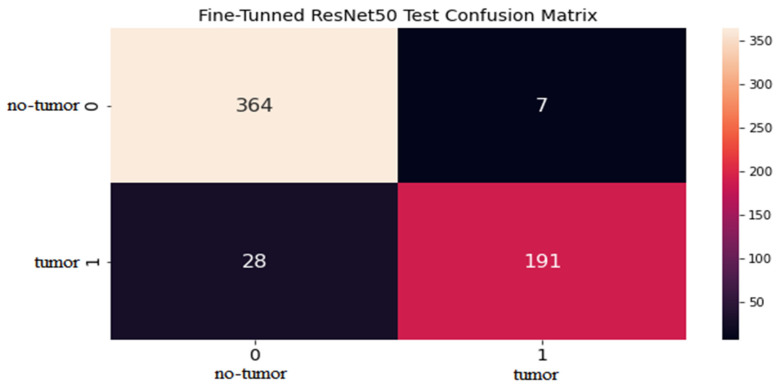
Test confusion matrix for fine-tuned ResNet50 model.

**Figure 6:**
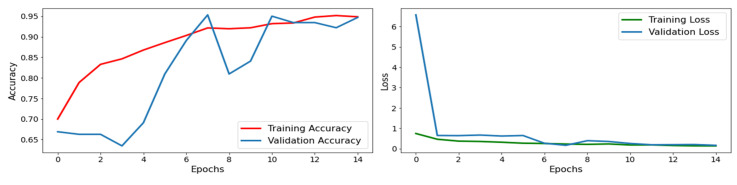
Accuracy and loss graph.

The proposed model uses the CNN model with fine-tuned ResNet50 architecture for brain tumor classification. The model is trained on the dataset of brain MRI scans with labelled tumor types, including tumor and non-tumor images. The results of the ResNet50 model for brain tumor classification demonstrate high accuracy, typically 94%, as shown in the confusion matrix in Figure 5. The graphical explanation of accuracy and loss is also shown in Figure 6 with red and blue lines. The model’s precision, which calculates the proportion of actual positive tumor type classifications out of all positive classifications, is also high, typically ranging from 93% to 96%. The recall, which measures the ratio of true positive tumor type classifications out of all actual tumor type cases, is also high, typically ranging from 87% to 98%. The F1 score, the harmonic mean of precision and recall, is also high, typically ranging from 0.92 to 0.95, as presented in Table 1.

On the other hand, the support values for tumor are 219 and for non-tumor are 371. The CNN model with fine-tuned ResNet50 architecture is an effective tool for brain tumor classification, achieving high statistical values. The model can help radiologists and clinicians to accurately identify the type of brain tumor from MRI images, leading to more personalized and targeted treatment plans for patients.

### 4.3. U-Net Model Results

**Figure 7:**
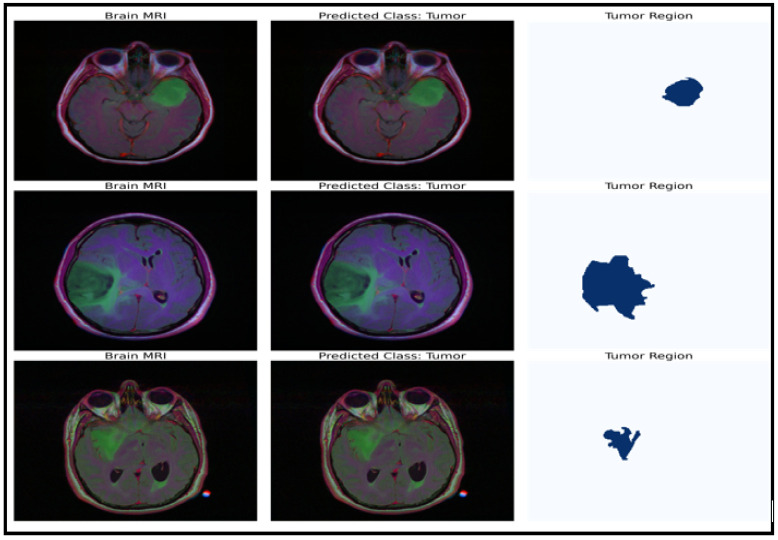
Dataset classification and model prediction of tumor regions.

**Figure 8:**
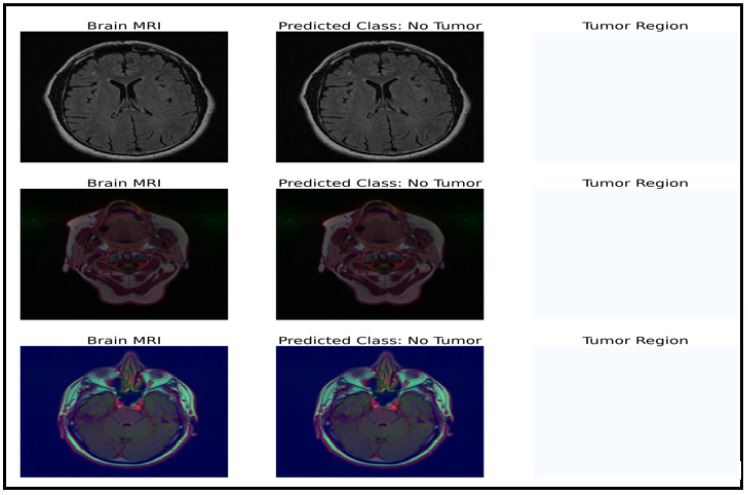
Dataset classification and model prediction of no-tumor regions.

The model can be used for brain tumor segmentation. The result of the U-Net architecture is a probability map representing the likelihood of each pixel in the input image belonging to the tumor region. A threshold can be set on this map to obtain the binary segmentation map, which indicates the presence or absence of the tumor in each pixel of the input image. The binary segmentation map obtained from the U-Net can be used to improve the accuracy of the brain tumor detection and classification tasks performed by the CNN model with fine-tuned ResNet50. For example, the binary segmentation map can focus the proposed model’s attention on the tumor region, reducing false positives and improving the overall accuracy. To integrate the outputs from the CNN model with fine-tuned ResNet50 and the U-Net architecture, the binary segmentation map can be overlaid onto the input MRI image to highlight the tumor region. Figure 7 shows the segmentation results for a selected tumor region, while Figure 8 shows the results for a non-tumor region. This combined approach can provide valuable insights for clinical diagnosis and treatment planning.

Focal Tversky Loss and Focal Tversky Accuracy are evaluation metrics modified from the standard Tversky Loss and Tversky Accuracy functions. Abraham et al. proposed them in a research paper titled “Focal Tversky loss function with improved Attention U-Net for lesion segmentation” [40]. 

**Figure 9:**
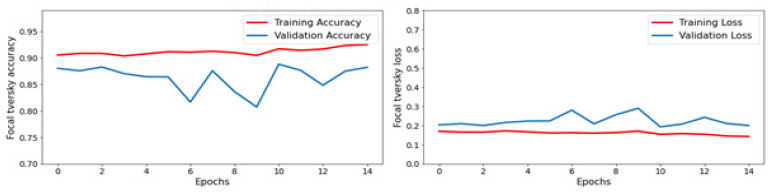
Focal Tversky Accuracy and Loss.

Focal Tversky Loss is formulated as (1—Tversky Index)^γ, where γ is a hyperparameter called the “focusing parameter” that controls the weight of false positives and false negatives. Compared to the standard Tversky Loss, Focal Tversky Loss penalizes false positives and negatives more heavily, making the model more sensitive to detecting small and rare classes, as shown in Figure 9.

Focal Tversky Accuracy is formulated as Tversky Index^γ, where γ is the same focusing parameter as in Focal Tversky Loss. Focal Tversky Accuracy is similar to the standard Tversky Accuracy but is better suited for evaluating the performance of models on small and rare classes, as shown in Figure 9.

Overall, these modified evaluation metrics have been shown to improve the performance of models in image segmentation tasks, including brain tumor segmentation.

### 4.4. Evaluation Criterion

The evaluation criteria for detection and classification include many standard statistical metrics below. Here are Equations (5)–(10) for the evaluation criteria commonly used for this work:

**True positive (Tp):** This indicates the number of correctly recognized positive samples (with tumors) in the dataset.

**False positive (Fp):** This value shows the numeral of incorrectly identified positive samples (samples without tumors) in the dataset.

**True negative (Tn):** This shows the number of correctly identified negative samples (samples without tumors) in the dataset.

**False negative (Fn):** The sum of incorrectly identified negative samples (with tumors) in the dataset.

**Accuracy:** The ratio of correctly known samples in the dataset as given in Equation (5).

(5) $$Accuracy=\frac{\left(Tp+Tn\right)}{\left(Tp+Fp+Tn+Fn\right)}$$

**Precision:** The ratio of correctly identified positive samples out of all those identified as positive as given in Equation (6).

(6) $$Precision=\frac{Tp}{\left(Tp+Fp\right)}$$

**Recall:** The ratio of accurately identified positive samples out of all the actual positive samples as given in Equation (7).

(7) $$Recall=\frac{Tp}{\left(Tp+Fn\right)}$$

**F1 Score:** The harmonic mean of precision and recall contributes equivalent weight to both measures as given in Equation (8).

(8) $$F1-score=\frac{2\times \left(Precision\times Recall\right)}{\left(Precision+Recall\right)}$$

**Dice Similarity Coefficient:** The dice coefficient is comparable to the IoU. Since they are positively correlated, if one states that model A is better than model B in picture segmentation, the other will also state the same. They run from 0 to 1, with 1 being the highest similarity between the predicted and the truth, similar to the IoU. This statistical validation parameter evaluates the effectiveness of automated probabilistic fractional segmentation of MR images and the reliability of manual segmentations for spatial overlap correctness.

(9) $$DSC=\frac{Tp}{\frac{1}{2}\left(2Tp+Fp+Fn\right)}$$

**Intersection over Union (IoU):** An object detector’s accuracy on a particular dataset is evaluated using an evaluation measure known as intersection over union. The term derives from considering model positives (true positives and false positives) as one set, and the dataset classification positives (true positives and false negatives) as another set. The intersection of these two sets is *Tp*, the union is *Tp* + *Fp* + *Fn*. Intersection over Union (IoU) is the ratio of these numbers.

(10) $$IoU=\frac{Tp}{Tp+Fp+Fn}$$

**Similarity index (SI):** The SI is a crucial metric that quantifies the accuracy of tumor detection models. It measures the similarity between ground truth annotations and the model’s segmentation output. A higher SI value indicates a more robust match between the predicted segmentation and the ground truth, reflecting more accurate tumor region detection.

(11) $$SI=\frac{2Tp}{2Tp+Fp+Fn}$$

These evaluation criteria help to assess the performance of brain tumor detection and classification models by measuring their ability to correctly identify positive and negative samples and differentiate between different types of tumors as shown in Table 2. They are commonly used in research studies and clinical practice to evaluate these models’ effectiveness and compare them against other approaches.

## 5. Discussion

In integrating U-Net with Resnet 50, the dice coefficient value is 0.95. The value of IoU is 0.91, and SI is 0.95, as shown in Table 3. Table 2 in this study compares the performance of two different models, CNN and fine-tuned ResNet50, in classifying brain tumors from MRI scans. The results show that the Fine-Tuned ResNet50 model outperforms the CNN model regarding precision, recall, F1 score, and accuracy for non-tumor and tumor classes. For the non-tumor class, the fine-tuned ResNet50 model achieved a precision of 0.98, recall of 0.95, F1 score of 0.93, and accuracy of 0.94, indicating that it correctly classified a high percentage of non-tumor cases.

Similarly, for the tumor class, the fine-tuned ResNet50 model attained a precision of 0.87, recall of 0.92, F1 score of 0.88, and accuracy of 0.96, indicating that it correctly classified a high percentage of tumor cases. Overall, these results suggest that the fine-tuned ResNet50 model is a more effective tool for identifying brain tumors from MRI images, which could lead to more accurate and personalized treatment plans for patients. The results from this work are IoU:0.91, DSC:0.95, SI:0.95, and have essential associations for the field of medical image analysis and could potentially improve patient outcomes. One potential limitation of the proposed study is the reliance on publicly available datasets (TCGA-LGG and TCIA), which may not fully capture the diversity of brain tumor cases and could introduce biases in the results. Additionally, the evaluation of the models’ performance metrics may not fully reflect real-world clinical scenarios, warranting further validation on larger and more diverse datasets.

## 6. Conclusions

This study proposed a CNN model with fine-tuned ResNet50 and U-net for brain tumor classification and detection in MRI images. This model combines the strengths of two different architectures to achieve high accuracy in both tasks. The Fine-Tuned ResNet50 architecture is used for brain tumor detection, which involves identifying the presence of a tumor in MRIs. The U-net architecture is used for brain tumor segmentation, which involves accurately delineating the tumor from the surrounding healthy tissue. The study compared the performance of two models, CNN and fine-tuned ResNet50, for brain tumor classification and detection using MRI images. The results indicate that the fine-tuned ResNet50 model outperforms the CNN model regarding statistical values for non-tumor and tumor classes. The fine-tuned ResNet50 model attained a precision of 0.98, recall of 0.95, F1 score of 0.93, and accuracy of 0.94 for the non-tumor class, and a precision of 0.87, recall of 0.92, F1 score of 0.88, and accuracy of 0.96 for the tumor class. The results from fine-tuned ResNet50 model are IoU:0.91, DSC:0.95 SI:0.95. These findings suggest that the fine-tuned ResNet50 model can be a valuable tool for accurately detecting and classifying brain tumors using MRI images.

## Figures and Tables

**Table 1:** Description of CNN layers.

Layer Name	Description
Image input	It takes the image as input with width, height, and color channel
Convolutional	It is the fundamental part of CNN and is used for feature extraction.
Batch Normalization	This layer provides zero input as mean and variance values, strengthening the network.
ReLU	It provides nonlinear values as input and zeros all the negative and odd values.
Pooling	It controls overfitting, handles the parameters, and is used after every Convo layer in a convolutional neural network. This layer is used in three forms, min, max, and average pooling layer.
Softmax	This layer uses processed data from the pooling and convolutional layer and feeds them as an initial value to CNN.
Fully Connected Layer	This layer performs actual classification by taking all the inputs to the final network. It is connected to all the neurons of the neural network for classification purposes.
Classification Layer	This layer computes the actual class entropy loss values for multiple class problems and, in the end, matches the classes with their actual class category.

**Table 2:** All model results.

Class Name	CNN Model					Fine-Tuned ResNet50 Model					U-Net Fine-Tuned Resnet50 Model		
	Precision	Recall	F1-Score	Support	Accuracy	Precision	Recall	F1-Score	Support	Accuracy	DSC	IOU	SI
0 (Non-tumor)	0.90	0.97	0.93	371	0.92	0.93	0.98	0.95	371	0.94	0.95	0.91	0.95
1 (Tumor)	0.94	0.83	0.88	219		0.96	0.87	0.92	219				

**Table 3:** Statistical values of U-Net with fine-tuned Resnet50 model.

Reference	Segmentation Technique	Dataset	Results
[41]	Multi-level threshold technique	BRATS 2015	DSC 0.89
[42]	K-means	BRATS 2017	SI 0.91
[43]	Random	BRATS 2012	DSC 0.91
[44]	Darwinian Particle Swarm Optimization	MRI images	DSC 0.93
[45]	Morphological technique	MRI images	DSC 0.90
**Our method**	**U-Net with fine-tuned Resnet50**	TCGA-LGG and TCIA	IoU 0.91, DSC 0.95, SI 0.95

## Data Availability

Dataset is publicly available and can be downloaded from the given link: https://wiki.cancerimagingarchive.net/pages/viewpage.action?pageId=5309188 (accessed on 10 May 2023).
